# Effect of utilizing a *Bacillus sp* bacteria consortium for biofilter inoculum in a lab-scale tilapia RAS to enhance production and fish health

**DOI:** 10.1038/s41598-026-60272-9

**Published:** 2026-07-06

**Authors:** Mohamed M. El-Naghy, Samar A. Tawfik, Malik M. Khalafalla, Mohamed M. Zayed, Mohamed M. Abdel-Rahim

**Affiliations:** 1 Department of Aquaculture, Faculty of Aquatic and Fisheries Sciences, Kafrelsheikh University, Kafr El-Shaikh, Egypt; 2 Aquaculture Division, National Institute of Oceanography and Fisheries, NIOF, Cairo, Egypt

**Keywords:** Nile tilapia, RAS, MBBR, *Bacillus* spp. inoculation, Performance, Well-being, Biotechnology, Microbiology, Zoology

## Abstract

For the success of recirculating aquaculture systems (RASs), effective biological filtration is essential. This study examined the biofiltration efficiency of four moving bed biofilm reactor (MBBR; abbreviated BF) configurations inoculated with a *Bacillus spp*. consortium (BC) and their effects on tilapia performance and health. Four treatments were tested: (1) **BF**_**120**_**BC**_**0**_: Biofiltration process lasted 120 days, 60 days before and 60 days during the experiment, without *Bacillus spp.* consortium addition. (2) **BF**_**67**_**BC**_**67**_: Biofiltration process lasted 67 days;7 before and 60 during the experiment. The MBBR received 1 g BC/m^3^ media every 15 days. (3) **BF**_**120**_**BC**_**60**_: Biofiltration process lasted 120 days, with 60 days without *Bacillus spp.* Consortium addition before the experiment. Then, the MBBR received 1 g BC/m^3^ media every 15 days for 60 days. (4) **BF**_**120**_**BC**_**67**_: From day 60 to day 7 before the experiment, the MBBR’s media was half (2.5 L) and then full (5 L). The MBBR was given 1 g BC/m^3^ media every 15 days for 67 days, starting from day 7 before the experiment. Using 12 RAS units, 120 tilapia weighing 72.41 g were stocked at 10 fish per tank, totaling 12 kg/m^3^, in four groups of three replicates. The first month’s water exchange was 1%, the second 2%. The results showed that adding *Bacillus spp.* consortium to the longer MBBR configuration period improved water quality, fish performance, and tilapia health. Compared with the other treatments, the **BF**_**120**_**BC**_**60**_ treatment showed enhanced performance in tilapia growth, feed utilization, and body composition. It also had a significant (*P* ≤ 0.05) effect on serum digestive enzymes, lipid profiles, liver and kidney enzymes, immunity, stress levels, antioxidants, immune/growth-related hormones, and health indicators in the gills, intestines, and liver.

## Introduction

Global tilapia production was 6.549 million tons in 2022^1^ and is estimated to exceed 7 million metric tons in 2025. This growth is driven by a rebound from COVID-19-related disruptions and increased consumer demand, especially in domestic markets. China remains the largest producer, but other countries, such as Indonesia, Brazil, and Egypt, are also experiencing substantial growth^2^. The limited availability of freshwater resources suitable for cultivating tilapia and climate change are the main challenges to the sustainability of this industry in numerous countries^3,4^. Therefore, RAS technology may be a vital solution for the sustainability of the aquaculture industry in many countries around the world that face serious water challenges.

Recirculating aquaculture systems (RASs) are among the most successful production techniques for enhancing the sustainability of aquaculture production^5^. The scarcity of food, coupled with the increase in the human population, resource depletion, and environmental degradation, constitutes a significant issue for both industrialized and developing countries. The transition to a more sustainable food agenda is one of the Sustainable Development Goals (SDGs)^6^. To meet nutritional needs, improving food production efficiency and minimizing food waste are essential. By 2050, the aquaculture industry is expected to be the main contributor to seafood security worldwide^7^. The aquaculture sector varies from extensive to intensive systems on the basis of feed inputs, fertilizers, water-use efficiency, and the stocking density of cultured organisms^4,8^. The traditional aquaculture industry is rapidly advancing, generating a substantial amount of wastewater^9^. Recirculating aquaculture systems (RASs) are intensive aquaculture systems that exclude the cultivation environment from external conditions^5^. RAS has emerged as a widespread technology in response to the shortage of water resources and the challenges of environmental pollution management^10^. The key benefit of RAS is its capacity to decrease the requirement for fresh, potable water while maintaining a healthy ecosystem for fish. Sequential treatment techniques are implemented to sustain water quality^8^. These processes are frequently performed either sequentially or concurrently. The categorization is as follows: (i) solid waste removal, (ii) biofiltration for ammonia and nitrite removal, (iii) aeration/oxygenation, (iv) pH/temperature adjustment, and (v) disinfection^8,10,11^.

Biofilters are utilized in water recycling to eliminate fish feed waste, subsequently returning the cleaned water to the system, which constitutes 90–99% of the recycled water^8^. They undergo aerification and receive media to augment their surface area, promoting the proliferation of beneficial bacteria. Regulating the intricate interactions between a bacterial population and its environment, as well as among its members, poses a challenge for biofilters^12,13^. Biofilters are considered the fundamental component of the RAS; without a reliable and stable biofilter, the system is susceptible to failure or disturbance. Bacterial biofilms form in biofilter compartments and oxidize toxic metabolic byproducts, including ammonia and nitrite, that accumulate in recirculating aquaculture systems^14^. Aerobic nitrification is a technique that removes nitrogenous wastes aerobically via biofilters. It is a two-step process in which ammonia is converted to nitrite and then to nitrate. This is accomplished by two bacterial consortia, (i) ammonia-oxidizing bacteria (AOB) and (ii) nitrite-oxidizing bacteria (NOB), which are both aerobic lithoautotrophic bacteria. AOB consists of bacteria from two families: β-proteobacteria (e.g., *Nitrosomonas*, *Nitrosovibrio*, *Nitrolobus*, and *Nitrospira*) and γ-proteobacteria. NOB comprises bacteria from seven genera and four phyla: *Nitrobacter*, *Nitrotoga*, *Nitrospira*, *Nitrococcus*, *Nitrospina*, *Nitrolancea*, and *Candidatus Nitromaritima*^8,15^. Generally, the system requires a daily replacement of approximately 5% of its water, which equals 500–1000 L/kg of feed, to prevent the buildup of dissolved organic compounds and nitrates^16^. To reduce the system’s water demand, nitrogen waste must be eliminated^17^. The disposal of these waste products must be performed in a cost-effective and environmentally friendly manner^18^. A nitrifying biofilter precedes the exclusion of ammonia-related residues. A microbial consortium transforms ammonia into nitrate, a less harmful form, which water exchange then eliminates^8^. Biofilters help with other useful processes that keep RAS water quality satisfactory, such as autotrophic and sulfide-dependent denitrification^12^.

Recirculating Aquaculture Systems (RAS) have basic biological filters that include submerged static-bed, trickling, rotating biological contactor (RBC), and moving-bed biofilm reactor (MBBR) or fluidized-bed filters. These filters are grouped by the kind of media and flow configuration^19^. The MBBR filter possesses a minimal footprint, effectively degrades ammonia and nitrites, ease in operation, maintenance and simplicity, demonstrates operational stability, low sludge production, no need of periodic backwashing, low pumping costs, and requires low maintenance owing to the media’s self-cleaning properties^20^. The MBBR biofiltration technology has been implemented globally in large-scale commercial and ornamental aquaculture projects^21^.

The utilization of *Bacillus* spp. as a probiotic supplement in aquaculture increased the overall bacterial load, eliminated coliform bacteria, promoted beneficial lactic acid bacteria, and provided resistance against pathogens, including *Aeromonas hydrophila*, *Vibrio* spp.*,* and *Acinetobact*er^22^. In aquaculture, probiotics refer to beneficial microorganisms, such as bacteria, introduced via feed or the surrounding environment. Probiotics can benefit the host by enriching the microbiome, ameliorating health problems, increasing growth efficiency, increasing feed intake, increasing stress responses, increasing disease resistance, and promoting general health^23^**.** *Bacillus* spp. are utilized to increase water quality and remediate various pollutants^24^. Moustafa, et al.^25^ revealed that synbiotics containing the probiotic *Bacillus subtilis* in fish diets enhanced growth by augmenting the immune response, antioxidative activity, and water quality. They are economical, stable, uncomplicated, spore-forming, and readily manufactured probiotics^26^**.** *Bacillus* spp. possess beneficial attributes such as stability across extreme temperatures, extended shelf life, resilience at elevated pH levels, and resistance to UV radiation, in addition to their established traits^23^.

The slow proliferation of existing nitrifying bacteria results in diminished efficiency of biofilters during the activation period^27^. The selection of an appropriate biofilter affects both capital and operational costs in an RAS system, enhances water treatment efficacy and overall water quality^28^, and promotes the proliferation of nitrifying populations to minimize the activation period. An ideal biofilter model would eliminate ammonia, minimize toxic nitrite levels, promote denser beneficial nitrifying bacterial species, and require inexpensive materials and upkeep to install. This biofilter can greatly improve aquaculture sustainability.

One significant aspect that lacks consensus in research is the appropriate timing for the introduction of bacteria into biological filters. There has been no research on the best time to add Bacillus spp. to biological filters in RAS farms. Moreover, the influence of the biological filter configuration period on water treatment efficacy, fish performance, and health has yet to be investigated. This study investigated how the configuration period of biological filters and the timing of Bacillus spp. additions affect various metrics in recirculating aquaculture systems (RAS) farms for Nile tilapia. Key areas of focus included water quality, fish growth, feed efficiency, blood and chemical profiles, body composition, antioxidant levels, immune response, and organ health.

## Materials and methods

### Ethical approval

The study experiment received approval from the Kafrelsheikh University Institutional Animal Care and Use Committee (IACUC approval number KFS-IACUC/290/2025), Kafrelsheikh University, Egypt. All methods were performed in accordance with the relevant guidelines and regulations.

### Experimental site

The experiment was carried out at the Department of Aquaculture, Faculty of Aquatic and Fisheries Sciences, Kafrelsheikh University, Kafrelsheikh, Egypt.

### Experimental fish and rearing techniques

Nile tilapia fingerlings were sourced from a private hatchery in Kafrelsheikh, Egypt, after receiving permission to obtain fish samples from the respective owner and the Faculty of Aquatic and Fisheries Sciences to use in the experiment. Fingerlings were acclimated to laboratory conditions featuring a light‒dark photoperiod cycle of 12 h each. Upon reaching a weight of 72.41 ± 0.14 g, 120 fish were allocated into four groups, each with three replicates. The fish were allocated at a density of 10 fish per tank; each tank contains 60 L of water (12.07 kg/m^3^), with every three RAS units representing one treatment.

### A detailed design of the RAS experimental unit

**Fig. 1:**
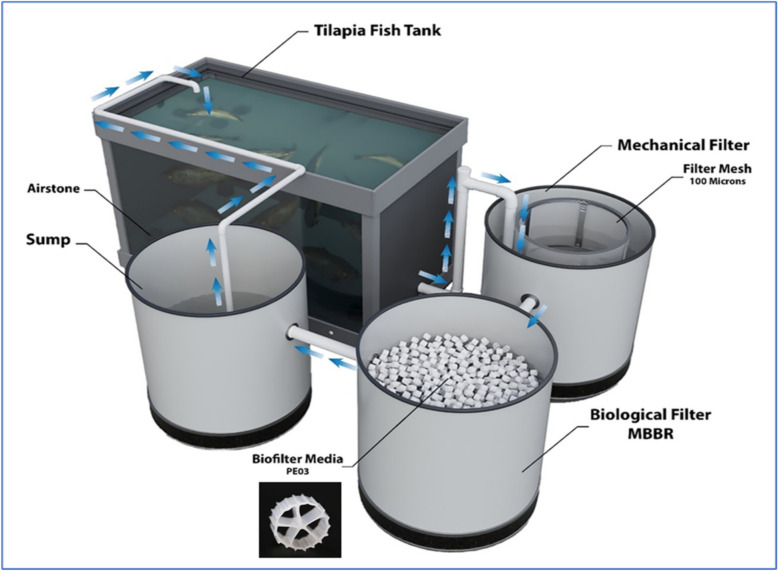
The design of the RAS unit used in the experiment. The experiment used 12 separate RAS units. Three RAS units represent one treatment, and a total of four treatments were evaluated.

Figure 1 illustrates the configuration of the RAS unit employed in the experiment. The experiment employed 12 separate RAS units, configured as outlined below:

**The fish tanks** were rectangular containers of 54 cm × 38 cm × 29.5 cm, with a capacity of 60 L. A bottom drain was installed with a 6% slope, and a standpipe was positioned externally to maintain the water level in the tank. The fish tank was aerated by four air stones positioned at the bottom.

Mechanical filters, biological filters, and sumps were cylindrical tanks measuring 25 cm in diameter and 24.5 cm in height, with a capacity of 12 L.

**Mechanical filters:** Filtration was performed using a 100-micron screen situated within the tank to segregate all solid waste prior to its discharge from the mechanical filter tank.

**Biological filters:** The biofiltration process was conducted via moving bed biofilm reactor (MBBR) technology. Each MBBR tank contained 5 L of PE03 HDPE biofilter media, produced by Tongxiang Small Boss Special Plastic Products Co., Ltd. (431 Tong Sheng Lu, Tong Xiang Shi, Jia Xing Shi, Zhe Jiang Sheng, China, 314,500), to promote bacterial proliferation. Five liters of PE03 HDPE media weigh 0.7 kg and provide a surface area of 5.0 m^2^. The media specification has a surface area of 1000 m^2^/m^3^, a diameter of 10 mm, a height of 7 mm, 5 holes, and a density of 0.94–0.97 g/cm^3^. Water infiltrated the MBBR biofilter tanks from the bottom and exited from the top. Two airstone diffusers are fixed to the tank’s bottom, facilitating aeration and mixing. The dimension is a 2" diameter spherical air stone. It is a medium-bubble air stone. Steady Output: Jetting volume: 5 L/min; Produced by the Pawfly company; Model: Pawfly Air Stone 2 Inch, Yuncheng Street, Room 411, No. 54, Xiaogang Garden Road, Guangzhou, Baiyun District, Guangdong, China 510,410.

**A sump tank:** It collects water from the MBBR unit for repumping to the fish tank at a rate of 60 L per hour via a submerged pump.

**Aeration:** Aeration was conducted using a 0.32 kW ring blower connected to several airstone diffusers.

**The *Bacillus spp.* Consortium (BC)** employed in the MBBR of this study was purchased commercially as Sanolife PRO-W® (INVE Company, Belgium). The product contains *Bacillus subtilis* and *Bacillus licheniformis* at a concentration of 2 × 10^11 cfu/gram, applied at a rate of 1 g/m^3^.

### Experimental design

Table 1 shows the experimental design and the timing of the *Bacillus* spp. consortium *(BC)* application. Twelve separate RAS units were used, with three of them representing one treatment.

**Table 1:** Experimental design indicating the timing of *Bacillus spp*. consortium (BC) incorporation into the MBBR (biofilter, BF) media.

Days before/after tilapia stocking	Treatments							
	BF_120_BC_0_		BF_67_BC_67_		BF_120_BC_60_		BF_120_BC_67_	
	MBBR media (Presence/kg)	*Bacillus spp*. Addition	MBBR media (Presence/kg)	*Bacillus spp*. Addition	MBBR media (Presence/kg)	*Bacillus spp*. Addition	MBBR media (Presence/kg)	*Bacillus spp*. Addition
**Day -60**	✓/0.70	−	−	−	✓/0.70	−	✓/0.35	−
**Day -45**	✓/0.70	−	−	−	✓/0.70	−	✓/0.35	−
**Day -30**	✓/0.70	−	−	−	✓/0.70	−	✓/0.35	−
**Day -15**	✓/0.70	−	−	−	✓/0.70	−	✓/0.35	−
**Day -7**	✓/0.70	−	✓/0.70	+	✓/0.70	−	✓/0.70	+
Day 0*	✓/0.70	−	✓/0.70	+	✓/0.70	+	✓/0.70	+
**Day + 15**	✓/0.70	−	✓/0.70	+	✓/0.70	+	✓/0.70	+
**Day + 30**	✓/0.70	−	✓/0.70	+	✓/0.70	+	✓/0.70	+
**Day + 45**	✓/0.70	−	✓/0.70	+	✓/0.70	+	✓/0.70	+
**Day + 60**	✓/0.70	−	✓/0.70	+	✓/0.70	+	✓/0.70	+

* Day 0 = the day of tilapia stocking; BC = *Bacillus spp*. Consortium; BF = MBBR biofilter; ( +) = the addition of *Bacillus spp*. Consortium at 1 g BC/m^3^ of media every 15 days; (-) = No addition of *Bacillus spp*. Consortium; (✓/0.70) = the presence of biological filter’s media and the quantity per biofilter (5 L, 0.70 kg); (✓/0.35) = the presence of biological filter’s media and the quantity per biofilter (2.5 L, 0.35 kg).

Four treatments were tested to assess how the addition of *Bacillus spp.* consortium affects the MBBR biofilter in a functioning RAS, detailed as follows:

**BF**_**120**_**BC**_**0**_**:** Control group. The biofiltration process lasted 120 days, 60 days before the experiment began and 60 days after the trial ended, without any *Bacillus spp.* consortium additions to the MBBR biofilter.

**BF**_**67**_**BC**_**67**_**:** The biofiltration process lasted 67 days, with 7 days before the experiment and 60 days during it. The biofilter received 1 g BC/m^3^ of media every 15 days.

**BF**_**120**_**BC**_**60**_**:** The biofiltration process lasted 120 days, with 60 days without *Bacillus spp.* consortium before the start of the experiment. Thereafter, the MBBR was given 1 g BC/m^3^ of media every two weeks for 60 days.

**BF**_**120**_**BC**_**67**_**:** From day 60 to day 7 before the experiment, the biological filter’s media volume was half its capacity (2.5 L, 0.35 kg) and then full (5 L, 0.70 kg). The MBBR was given 1 g BC/m^3^ of media every two weeks for 67 days from day 7 prior to the start of the experiment until its conclusion.

During the start-up period, a small amount (0.5 g) of commercial fish feed (30% protein) was added every 15 days to the biological filter as an organic nitrogen source. Decomposition of the feed generated ammonia to support the natural fixation of nitrifying bacteria within the biofilter. No ammonium formate or other inorganic ammonia sources were added to the system during the biofilter preparation. This feed-based start-up approach for 2 months before starting the experiment allowed gradual biofilm formation on the media and stabilization of the biofiltration process before the experimental treatments were initiated^29^. The duration of the experimental period was 60 days. During the experimental period, Nile tilapia were raised in RAS using dechlorinated tap water as a water supply source. Dechlorination was conducted with sodium thiosulfate (1 g/10 l of water). The daily water exchange rate was almost 1% during the first month and increased to 2% during the second month to reduce nitrate levels and improve water clarity.

### Diet preparation and feeding regime

Table 2 presents the formulation and chemical composition of the experimental diet. During the trial, the fish were fed a formulated diet containing 30% protein and 7% fat at a rate of 3% of their total biomass, distributed in four meals daily, seven days a week. Fish samples were collected weekly to recalculate the feeding quantity.

**Table 2:** Chemical composition of the experimental diet.

Ingredients	g/kg	Parameter	g/100 g
Fish meal (62%—Vietnam)	100	Moisture	7.6
Gluten (corn)	90	Protein	29.7
Soybean meal (46-expel)	350	Fat	7.2
Rice bran	100	Ash	9.6
Corn (7.5% CP)	246	Fiber	4.6
Fish oil	10	NFE^1^	41.3
Soybean oil	20	Gross energy (MJ/kg)^2^	16.92
Trace mineral premix	5		
Vitamin premix	5		
Dicalcium Phosphate	12.5		
Bone meal	20		
Corn/wheat starch	20		
Vitamin C	5		
Salt (NaCl)	10		
Lysine (L-LYS HCL)	2.5		
Probiotics	2		
Antitoxins (Zeolite)	2		
Total	1000		

1 Nitrogen free extract (NFE, %) = 100 − (moisture + crude protein + crude lipids + fibers + ash).

2 Gross energy (GE, MJ/kg)) was calculated as 23.4, 39.8, and 17.2 kJ/g for protein, lipids, and carbohydrates, respectively.

### Water quality assessment

Water quality parameters were monitored throughout the experimental period. Dissolved oxygen (DO) levels were measured daily via the MW600 (mg/l) produced by Milwaukee Instruments, Inc. (2950 Business Park Drive, Rocky Mount, NC 27,804 United States). Water temperature (°C) and pH were monitored daily with HANNA HI98107. The TDS was measured via a HANNA HI98130. Total ammonia nitrogen (TAN) was measured twice a week via a HANNA HI-97715 medium-range ammonia portable photometer (HANNA Instruments, Romania). Unionized ammonia (NH₃) was subsequently calculated via temperature, pH, TAN, and salinity data. Nitrite, nitrate, and alkalinity levels were measured twice a week via a HANNA HI83300-02 multiparameter benchtop photometer and pH meter. NO₂ was measured using the Hanna Instruments Low Range Portable Photometer Kit—HI-97707C, while NO₃ was measured using the Hanna Instruments HI97728 Nitrate Photometer produced by Hanna Instruments, Inc. (584 Park East Drive, Woonsocket, RI 02,895, USA).

### Fish and feed analytical methods

The chemical compositions of the prepared diet and the fish were assessed at the start of the experiment with ten fish and at the end with nine fish per treatment (three per replicate) to evaluate moisture, crude protein, crude fat, fiber, and ash contents, in accordance with the Association of Official Analytical Chemists^30^. The gross energy (GE) values were determined to be 23.4 kJ/g for protein, 39.8 kJ/g for lipids, and 17.2 kJ/g for carbohydrates, respectively^31^.

### Fish sampling, growth, feed utilization and somatic indices

At the onset of the trial, each fish in the tanks was weighed individually, followed by biweekly weighing afterwards. The feed amount (FI) provided to each tank was modified based on the total fish biomass. At the conclusion of the experiment, the weights and numbers of fish in each experimental tank were employed to evaluate the growth performance, feed efficiency, survival rate, condition factor, viscera-somatic index, and hepatosomatic index of Nile tilapia using the following equations:

1. Weight gain (g/fish) = final body weight − initial body weight.
2. Average daily gain (ADG, g/fish/day) = weight gain/ experimental days.
3. Specific growth rate (SGR, %) = 100 × (ln (final body weight)—ln (initial body weight))/experimental days.
4. Feed intake (FI, g/fish) = feed consumption (g)/average number of fish at the end.
5. Feed conversion ratio (FCR, g) = total feed consumption (g)/total weight gain (g).
6. The protein efficiency ratio (PER, %) = 100 × (total weight gain (g)/protein intake (g)).
7. Protein productive value (PPV, %) = 100 × (protein gain (g)/protein intake (g)).
8. Energy gain (EG, Kcal) = Energy content in the fish carcass (Kcal) − Energy content in the fish carcass (Kcal) at the end
9. Energy utilization (EU, %) = 100 × (energy gain (kcal)/energy intake (kcal).
10. Condition factor (CF) = final body weight (g)/final body length (cm)^3^.
11. Hepatosomatic index (HSI, %) = 100 × (liver weight (g)/fish body weight (g)).
12. Viscera-somatic index (VSI, %) = 100 × (viscera weight (g)/fish body weight (g)).

### Hematological biochemical analysis

#### Blood sampling

At the end of the experiment, blood samples (three fish per replicate) were drawn from the caudal vein of an anesthetized fish (0.3 ml/l clove oil prior to blood collection) via a 3 mL medical syringe. The blood was divided into two tubes, one of which contained EDTA (1 mg/ml) as an anticoagulant for hematological investigation. The second sample was obtained by centrifuging the coagulated blood at 4000 rpm for 15 min at 4 °C without anticoagulant and was then stored at -20 °C until analysis.

#### Blood analysis

An automatic blood cell counter (Exigo-Vet, Boule Medical AB Inc., Stockholm, Sweden) was used to measure the number of red blood cells (RBCs), hemoglobin concentration, packed cell volume (PCV), and total and differential white blood cells (WBCs)^32^.

### Serum biochemical analysis

#### Serum digestive enzymes, lipid profile, and stress markers

The activities of amylase, protease, and lipase were assessed according to the methodologies described by Zamani, et al.^33^. The total cholesterol concentration is ascertained via free cholesterol and cholesteryl ester enzyme tests^34^. The serum triglyceride (TG) concentration was assessed via a TG quantification kit (MAK266, Sigma‒Aldrich, St. Louis, MO, USA). In this assay, triglycerides are converted to free fatty acids and glycerol^35^. The levels of high-density lipoprotein (HDL) and low-density lipoprotein (LDL) were assessed via the techniques established by Vassault^36^. Serum alanine aminotransferase (ALT) and aspartate aminotransferase (AST) levels were quantified via the method described by Bergmeyer, et al.^37^; 0.2 M DL-aspartic acid and 20 mM L-ketoglutarate were used as substrates for ALT, and 0.2 M DL-alanine and 2 mM L-ketoglutarate were used for AST. The activity of alkaline phosphatase (ALP) was evaluated via an enzymatic colorimetric method as outlined by Tietz, et al.^38^. The measurement technique outlined by Whitehead, et al.^39^ was employed to assess the renal function indicators: urea, uric acid, and ammonia. Serum creatinine (CRE), glucose (GLU), and cortisol (COR), were evaluated via the methods described by Heinegård and Tiderström^40^, Trinder^41^, and Sadoul and Geffroy^42^, respectively.

### Immunity and antioxidant parameters

Total serum immunoglobulin (IgM) was quantified by precipitating immunoglobulin with polyethylene glycol and calculating the difference between the starting and final total protein levels, as described by Siwicki^43^. The total plasma protein concentration (g dL⁻^1^) was evaluated via the biuret method as described by Doumas, et al.^44^. The albumin concentration (g dL⁻^1^) was evaluated via the bromocresol green method as described previously by Reinhold^45^, whereas the globulin concentration (g dL⁻^1^) was calculated by subtracting the albumin concentration from the total protein concentration. The serum complement 3 (C3) levels were assessed via the immunological turbidimetric technique outlined by Zhou, et al.^46^. The total antioxidative capacity (TAC) was evaluated via the method of Koracevic, et al.^47^ with a Hitachi 7600D analyzer (Hitachi, Tokyo, Japan). The catalase (CAT) enzyme was measured via the method developed by Aebi^48^. Malondialdehyde (MDA) levels were evaluated according to the approach established by Uchiyama and Mihara^49^. Superoxide dismutase (SOD) was assessed following the methodology of Misra and Fridovich^50^. Glutathione peroxidase (GPx) levels were quantified via the method of Paglia and Valentine^51^.

### Growth and immunity hormones

Growth hormone (GH), insulin-like growth factor 1 (IGF-1), and B-actin were evaluated according to procedures developed by Cusabio Company (7505 Fannin St., Ste 610, Room 7 (CUBIO Innovation Center), Houston, TX 77,054, USA). The quantification of GH in fish plasma was performed in accordance with the manufacturer’s guidelines with fish-specific ELISA-GH kits (https://www.cusabio.com/uploadfile/Ins/CSB-E12121Fh.pdf). Furthermore, IGF-1 concentrations in fish plasma were quantified with fish-specific IGF-1 ELISA kits (https://www.cusabio.com/uploadfile/Ins/CSB-E12122Fh.pdf). Additionally, serum beta-actin (B-actin) was quantified via a fish ELISA kit for B-actin according to the manufacturer’s guidelines (https://www.cusabio.com/uploadfile/Ins/CSB-E62971Fh.pdf).

### Histopathological procedures

**Histopathological analysis:** Fish were anesthetized using 0.3 ml/l clove oil prior to histopathological analysis. This concentration has less effect on the morphology of the gills. Fish samples were obtained from the gills, liver, and middle intestine. For gill histology, we carefully open the operculum and use sharp surgical scissors and fine scissors to cut the gill arch from the top and bottom, avoiding touching the filaments with the cutting tool, as they are very delicate. The second or third gill arch from the right or left side was removed to standardize the samples. Then, samples were promptly taken from the dissected fish and subsequently immersed in a 10% buffered formaldehyde solution for fixation. Then tissues transferred to 70% ethyl alcohol until histological preparations (cutting, staining) are performed. Tissues were imbedded in paraffin wax following dehydration and clearing. Sections of 5 μm thickness were prepared via a Leica Rotary Microtome (RM 2145, Leica Microsystems, Wetzlar, Germany). The serial sections were deparaffinized by sequentially immersing them in xylene, followed by alcohol and finally water for staining. Each slide was stained with hematoxylin and eosin (H&E) according to the protocol established by Bancroft and Layton^52^. Histomorphometry was conducted via ImageJ analysis software (National Institutes of Health, MD, USA) to examine the presence, distribution, and infiltration of inflammatory cells or alterations in the fish tissues. The dimensions of the intestinal villi, including length, width, and intervilli spacing, were quantified via ImageJ analysis software and are expressed in micrometers (μm) (https://imagej.nih.gov/ij/download.html).

### Sampling, enumeration and identification procedures for major pathogenic bacteria

Prior to the conclusion of the trial (day 60), water samples for quantifying pathogenic bacteria in the experimental units were collected via previously defined protocols^53^. Samples in bottles stored in darkness at a consistently low temperature. Water samples were collected in sterile 500 mL screw-cap bottles, as recommended by Austin^54^. For culturing, 1 mL of each sample was diluted in 99 mL of saline and cultured via the pouring plate technique on nutrient agar medium at 30 °C for 18–24 h. The quantities of microorganisms evaluated were denoted as CFU/100 mL. Bacterial testing was conducted via the membrane filtration methods outlined in ISO 9308/1 (2000) and 7899/2 (2000). The filtered diluted samples (1 mL) were passed through a 0.45 µm pore size (47 mm diameter) sterile cellulose membrane grid. Fecal *Streptococcus* membranes were inoculated into Slanetz and Bartley media following 72 h of incubation at 37 °C. The dark red colonies were enumerated to determine *Vibrio* species. The membranes were inoculated onto thiosulfate citrate bile salt sucrose (TCBS) agar and incubated at 37 °C for 24 h. Extensive green and/or yellow colonies were identified as *Vibrio* sp. *Salmonella* species, which were detected and quantified on bismuth sulfite agar and Salmonella-Shigella (SS) agar. The black colonies were enumerated following 18–24 h of incubation at 35–37 °C. *Aeromonas* species were identified via Aeromonas isolation medium. Following 18–24 h of incubation at 35–37 °C, opaque dark green colonies with dark centers were enumerated. The membranes were applied to mannitol salt agar for the detection and enumeration of *S. aureus*, followed by incubation at 35 °C for 18–24 h. The yellow colonies were subsequently enumerated. The membranes were placed on Pseudomonas isolation agar to detect and quantify *Pseudomonas* sp. Blue‒green colonies emerged after 40 to 48 h of incubation at 35 °C.

### Statistical analysis

The results for each assessed parameter are expressed as the mean ± standard error of the mean (SEM). The data satisfy the assumption of homogeneity of variance. The data were statistically analyzed via one-way analysis of variance (ANOVA) with SPSS software (Standard Version 22.0, SPSS Inc., Chicago, Illinois). Comparisons of treatment means were conducted at the *P* ≤ 0.05 significance level via Duncan’s multiple range test. The Shapiro‒Wilk test was used to evaluate the normality of the collected data^55^. Figures were generated via GraphPad Prism 10 (GraphPad Software, San Diego, CA, USA).

## Results

### Water quality assessment

**Fig. 2:**
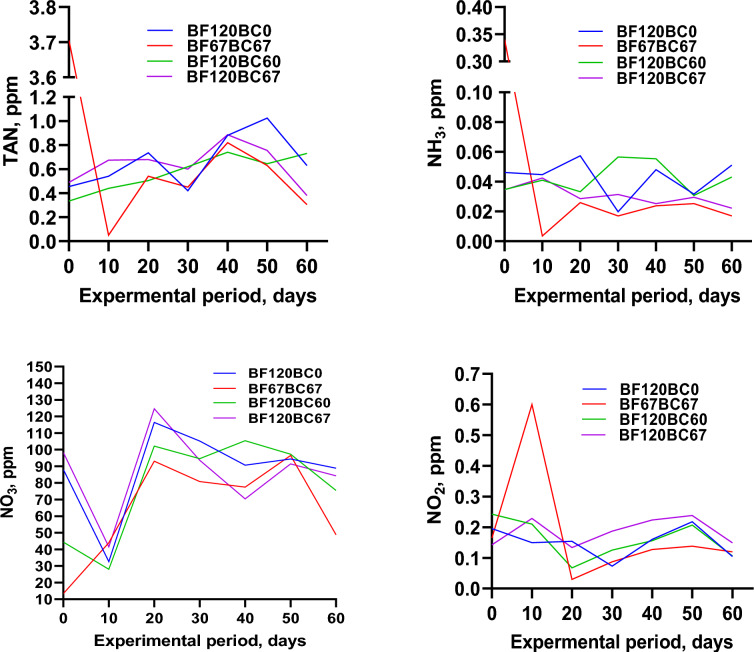
Periodical water quality parameters of Nile tilapia grown for 60 days in RAS-based tanks with diverse MBBR biofilter configurations; Treatments: (1) **BF**_**120**_**BC**_**0**_: The biofiltration process lasted 120 days, 60 days before and 60 days after the trial, without BC in the BF. (2) **BF**_**67**_**BC**_**67**_: Biofiltration process lasted 67 days;7 before and 60 during the experiment. The BF received 1 g/m^3^ BC every 15 days. (3) **BF**_**120**_**BC**_**60**_: Biofiltration process lasted 120 days, with 60 days without BC before the experiment. After that, the BF received 1 g BC/m^3^ media every 15 days for 60 days. (4) **BF**_**120**_**BC**_**67**_: From day 60 to day 7 before the experiment, the BF’s media was half (2.5 L) and then full (5 L). The BF was given 1 g BC/m^3^ of medium every 15 days for 67 days, starting from day 7 before the experiment. BF = MBBR Biofilter; BC = *Bacillus spp.* consortium; TAN, total ammonia nitrogen; NH_3_, unionized ammonia; NO_2_, nitrite; NO_3_, nitrate.

Table 3 presents the mean results of the water quality metrics throughout the study, whereas Fig. 2 illustrates the periodic findings for TAN, NH₃, NO₃, and NO₂ at 10-day intervals over the duration of the study. The statistical analysis results indicated no significant (*P* > 0.05) differences in the values of temperature, DO₂, TAN, NH₃, NO₂, and NO₃, but significant (*P* ≤ 0.05) changes were observed in the values of alkalinity, pH, and TDS. Severe fluctuations in the values of TAN, NH₃, NO₂, and NO₃ in **BF**_**67**_**BC**_**67**_ were evident throughout the first ten days of the experiment, in contrast to the relative stability of the other coefficients, as illustrated in Fig. 2.

**Table 3:** Water quality parameters of Nile tilapia grown for 60 days in RAS-based tanks with diverse MBBR biofilter configurations.

Parameters	Treatments*				*P*-Value
	BF_120_BC_0_	BF_67_BC_67_	BF_120_BC_60_	BF_120_BC_67_	
Temperature, ^o^c	25.85 ± 0.19	26.13 ± 0.14	26.04 ± 0.17	25.91 ± 0.18	0.656
DO_2_, ppm	6.12 ± 0.102	5.96 ± 0.080	6.0 ± 0.084	6.09 ± 0.104	0.614
pH	8.04 ± 0.05^ab^	7.91 ± 0.06^b^	8.11 ± 0.04^a^	7.91 ± 0.04^b^	0.013
TDS, ppm	0.74 ± 0.01^b^	0.56 ± 0.02^c^	0.81 ± 0.01^a^	0.72 ± 0.04^b^	0.001
Alkalinity, ppm	104.88 ± 4.06^b^	76.25 ± 2.75^c^	158.25 ± 4.82^a^	90.00 ± 8.69^bc^	0.001
TAN, ppm	0.67 ± 0.062	0.93 ± 0.321	0.57 ± 0.043	0.64 ± 0.051	0.461
NH_3_, ppb	40 ± 0.004	60 ± 0.031	40 ± 0.003	30 ± 0.002	0.498
NO_2_, ppm	0.15 ± 0.02	0.18 ± 0.05	0.16 ± 0.02	0.19 ± 0.02	0.788
NO_3_, ppm	88.01 ± 8.16	64.85 ± 8.22	78.19 ± 8.88	86.31 ± 7.58	0.189

Values in the same row with a different superscript are significantly different (*P* ≤ 0.05). Data are presented as (mean ± SEM) of 3 replicates. * Treatments: (1) **BF**_**120**_**BC**_**0**_: The biofiltration process lasted 120 days, 60 days before and 60 days after the trial, without BC in the BF. (2) **BF**_**67**_**BC**_**67**_: Biofiltration process lasted 67 days;7 before and 60 during the experiment. The BF received 1 g/m^3^ BC every 15 days. (3) **BF**_**120**_**BC**_**60**_: Biofiltration process lasted 120 days, with 60 days without BC before the experiment. After that, the BF received 1 g BC/m^3^ media every 15 days for 60 days. (4) **BF**_**120**_**BC**_**67**_: From day 60 to day 7 before the experiment, the BF’s media was half (2.5 L) and then full (5 L). The BF was given 1 g BC/m^3^ of medium every 15 days for 67 days, starting from day 7 before the experiment. DO_2_, dissolved oxygen; TDS, total dissolved solids; TAN, total ammonia nitrogen; NH_3_, unionized ammonia; NO_2_, nitrite; NO_3_, nitrate.

### Water microbiological analysis

Table 4 presents the average numbers of bacterial community data, including the total bacterial count; gram-negative bacilli; and *Streptococcus spp*. *Vibrio* spp. and *Aeromonas* spp. were not detected in the tank water, although other species were detected. Significant (*P* ≤ 0.05) differences in the total bacterial count, gram-negative bacilli, and *Streptococcus* spp. were detected among the treatments. Treatment **BF**_**120**_**BC**_**67**_ presented the highest total bacterial count and gram-negative bacilli values, whereas treatment **BF**_**120**_**BC**_**60**_ presented the highest values for *Streptococcus* spp.

**Table 4:** Water microbiological analysis of Nile tilapia grown for 60 days in RAS-based tanks with diverse MBBR biofilter configurations.

Bacterial community (CFU/100 mL)	Treatments*				*P*-Value
	BF_120_BC_0_	BF_67_BC_67_	BF_120_BC_60_	BF_120_BC_67_	
Total bacterial count	243.0 ± 11.00^c^	258.0 ± 5.00^c^	292.0 ± 6.00^b^	375.7 ± 9.39^a^	0.001
Gram negative bacilli	85.5 ± 5.50^b^	105.0 ± 5.00^b^	83.0 ± 7.00^b^	202.0 ± 5.77^a^	0.001
*Vibrio Spp*	0	0	0	0	–
*Aeromonas spp*.	0	0	0	0	–
*Streptococcus spp*.	40.0 ± 3.00^d^	59.0 ± 2.00^c^	130.5 ± 7.50^a^	94.0 ± 3.21^b^	0.001

The values with a different superscript in the same row are significantly different (p ≤ 0.05). * Treatments: (1) **BF**_**120**_**BC**_**0**_: The biofiltration process lasted 120 days, 60 days before and 60 days after the trial, without BC in the BF. (2) **BF**_**67**_**BC**_**67**_: Biofiltration process lasted 67 days;7 before and 60 during the experiment. The BF received 1 g/m^3^ BC every 15 days. (3) **BF**_**120**_**BC**_**60**_: Biofiltration process lasted 120 days, with 60 days without BC before the experiment. After that, the BF received 1 g BC/m^3^ media every 15 days for 60 days. (4) **BF**_**120**_**BC**_**67**_: From day 60 to day 7 before the experiment, the BF’s media was half (2.5 L) and then full (5 L). The BF was given 1 g BC/m^3^ of medium every 15 days for 67 days, starting from day 7 before the experiment.

### Growth performance, feed utilization, and chemical composition

Table 5 presents the results of growth performance, feed utilization, and biometric measurements. The findings of the present study revealed significant (*P* ≤ 0.05) changes in all the assessed parameters, except for energy utilization and K among the treatments. The best growth metrics, including final weight, ADG, and SGR, were observed in treatment **BF**_**120**_**BC**_**60**_, but treatment **BF**_**67**_**BC**_**67**_ presented the poorest values. Treatments **BF**_**120**_**BC**_**0**_ and **BF**_**120**_**BC**_**67**_ yielded comparable results, with no significant differences between them. An identical tendency is observed in the feed utilization results, particularly for FCR, PER, PPV, and energy gain. The chemical analysis of the fish indicated that there were no significant (*P* > 0.05) differences in dry matter, lipids, ash, or carcass energy among the treatments; however, there were significant (*P* ≤ 0.05) differences in protein content, with treatment **BF**_**67**_**BC**_**67**_ having the lowest protein level and **BF**_**120**_**BC**_**67**_ showing the highest (Table 6).

**Table 5:** Growth performance, feed utilization, biometric analyses, and body composition of Nile tilapia grown for 60 days in RAS-based tanks with diverse MBBR biofilter configurations.

Parameters	Treatments*				*P*-Value
	BF_120_BC_0_	BF_67_BC_67_	BF_120_BC_60_	BF_120_BC_67_	
Growth performance					
Initial weight, g/fish	72.25 ± 0.09	72.90 ± 0.40	72.20 ± 0.29	72.30 ± 0.06	0.250
Final weight, g/fish**	120.95 ± 2.74^ab^	114.40 ± 4.68^b^	126.85 ± 1.76^a^	124.60 ± 1.44^ab^	0.073
Weight gain (WG), g/fish**	48.70 ± 2.66^ab^	41.50 ± 5.08^b^	54.65 ± 1.47^a^	52.30 ± 1.39^a^	0.067
ADG, g/fish/day**	0.81 ± 0.04^ab^	0.69 ± 0.08^b^	0.91 ± 0.02^a^	0.87 ± 0.02^a^	0.067
SGR, %/day/fish**	0.86 ± 0.04^ab^	0.75 ± 0.08^b^	0.94 ± 0.02^a^	0.91 ± 0.02^ab^	0.068
Feed utilization					
Feed intake, g	84.10 ± 0.92^a^	79.70 ± 0.98^b^	83.60 ± 1.21^a^	84.57 ± 0.35^a^	0.021
FCR	1.74 ± 0.08^ab^	1.97 ± 0.22^a^	1.53 ± 0.02^b^	1.62 ± 0.04^ab^	0.127
PER, g/g	1.95 ± 0.08^ab^	1.75 ± 0.19^b^	2.20 ± 0.03^a^	2.08 ± 0.05^ab^	0.087
PPV, %	53.00 ± 4.28^ab^	45.61 ± 2.05^b^	56.52 ± 2.89^a^	57.68 ± 1.34^a^	0.064
Energy gain, Kcal	95.12 ± 5.77^ab^	82.59 ± 8.89^b^	103.50 ± 4.19^a^	97.13 ± 1.75^ab^	0.153
Energy utilization, %	25.89 ± 1.29	23.69 ± 2.29	28.35 ± 0.77	26.31 ± 0.42	0.208
Biometric indices					
CF	1.85 ± 0.03	1.90 ± 0.06	1.90 ± 0.02	1.93 ± 0.02	0.517
HSI	2.67 ± 0.15^ab^	1.85 ± 0.06^b^	2.56 ± 0.56^ab^	3.17 ± 0.47^a^	0.177
VSI	7.91 ± 0.06^b^	9.77 ± 0.01^a^	8.95 ± 0.59^ab^	10.22 ± 0.51^a^	0.014

Values in the same row with a different superscript are significantly different (*P* ≤ 0.05). Data are presented as (mean ± SEM) of 3 replicates. * Treatments: (1) **BF**_**120**_**BC**_**0**_: The biofiltration process lasted 120 days, 60 days before and 60 days after the trial, without BC in the BF. (2) **BF**_**67**_**BC**_**67**_: Biofiltration process lasted 67 days;7 before and 60 during the experiment. The BF received 1 g/m^3^ BC every 15 days. (3) **BF**_**120**_**BC**_**60**_: Biofiltration process lasted 120 days, with 60 days without BC before the experiment. After that, the BF received 1 g BC/m^3^ media every 15 days for 60 days. (4) **BF**_**120**_**BC**_**67**_: From day 60 to day 7 before the experiment, the BF’s media was half (2.5 L) and then full (5 L). The BF was given 1 g BC/m^3^ of medium every 15 days for 67 days, starting from day 7 before the experiment. ADG, average daily gain; SGR, specific growth rate; FCR, feed conversion ratio; PER, protein efficiency ratio; PPV, protein productive value; CF, condition factor; HSI, hepatosomatic index; VSI, viscerasomatic index. Average initial weight is (72.41 ± 0.14 g). ** Values for final weight, WG, ADG, and SGR with P-values between 0.067 and 0.073 are considered numerical trends and are not statistically significant at P ≤ 0.05.

**Table 6:** Chemical composition of Nile tilapia grown for 60 days in RAS-based tanks with diverse MBBR biofilter configurations.

Parameters	Initial	Final				*P*-Value
		BF_120_BC_0_	BF_67_BC_67_	BF_120_BC_60_	BF_120_BC_67_	
Dry matter, %	27.02 ± 0.57	32.13 ± 0.48	31.94 ± 0.37	31.80 ± 0.43	32.11 ± 0.44	0.039
Protein, %	59.67 ± 0.39	64.00 ± 0.85^ab^	61.82 ± 0.60^b^	63.64 ± 0.66^ab^	65.36 ± 0.60^a^	0.192
Lipids, %	24.61 ± 0.70	17.97 ± 1.27	19.50 ± 0.93	18.30 ± 0.74	16.11 ± 0.96	0.486
Ash, %	15.59 ± 0.21	18.03 ± 0.42	18.68 ± 0.34	18.06 ± 0.26	18.53 ± 0.36	0.941
Carcass E. Kcal/100 g	568.83 ± 0.56	530.59 ± 7.17	532.75 ± 5.41	531.68 ± 3.71	520.71 ± 5.68	0.451

Values in the same row with a different superscript are significantly different (*P* ≤ 0.05). Data are presented as (mean ± SEM) of 3 replicates. * Treatments: (1) **BF**_**120**_**BC**_**0**_: The biofiltration process lasted 120 days, 60 days before and 60 days after the trial, without BC in the BF. (2) **BF**_**67**_**BC**_**67**_: Biofiltration process lasted 67 days;7 before and 60 during the experiment. The BF received 1 g/m^3^ BC every 15 days. (3) **BF**_**120**_**BC**_**60**_: Biofiltration process lasted 120 days, with 60 days without BC before the experiment. After that, the BF received 1 g BC/m^3^ media every 15 days for 60 days. (4) **BF**_**120**_**BC**_**67**_: From day 60 to day 7 before the experiment, the BF’s media was half (2.5 L) and then full (5 L). The BF was given 1 g BC/m^3^ of medium every 15 days for 67 days, starting from day 7 before the experiment. Carcass E, carcass energy.

### Blood biochemical analysis

Table 7 presents the blood analysis findings for the tested treatments, revealing significant (*P* ≤ 0.05) variations among them. The key difference between the treatments was the elevated hemoglobin levels at **BF**_**120**_**BC**_**0**_, **BF**_**120**_**BC**_**60**_, and **BF**_**120**_**BC**_**67**_, whereas **BF**_**67**_**BC**_**67**_ had the lowest value. Furthermore, the WBC numbers were lower in **BF**_**120**_**BC**_**60**_ than in the other treatments and markedly elevated at **BF**_**120**_**BC**_**0**_ and **BF**_**67**_**BC**_**67**_. No significant changes were observed among the treatments for RBCs; however, substantial differences were noted in hematocrit values, with **BF**_**67**_**BC**_**67**_ exhibiting the lowest value.

**Table 7:** Blood biochemical analysis of Nile tilapia grown for 60 days in RAS-based tanks with diverse MBBR biofilter configurations.

Parameters	Treatments*				*P*-Value
	BF_120_BC_0_	BF_67_BC_67_	BF_120_BC_60_	BF_120_BC_67_	
Hemoglobin (g/100 ml)	9.14 ± 0.18^ab^	8.80 ± 0.15^b^	9.50 ± 0.12^a^	9.10 ± 0.06^ab^	0.039
RBCs (× 10^6^/mm^3^)	1.95 ± 0.05	2.09 ± 0.11	1.89 ± 0.09	1.90 ± 0.16	0.570
WBCs (× 10^3^/mm^3^)	94.00 ± 1.15^a^	90.00 ± 1.53^a^	78.00 ± 1.53^c^	85.00 ± 0.58^b^	0.001
Neutrophils (%)	33.00 ± 1.73	30.00 ± 1.53	35.00 ± 1.53	34.00 ± 1.15	0.182
Lymphocytes (%)	65.00 ± 2.08^a^	63.00 ± 1.73^a^	63.00 ± 2.08^a^	56.00 ± 1.00^b^	0.032
Monocytes (%)	1.00 ± 0.00^b^	5.00 ± 1.53^a^	1.00 ± 0.29^b^	8.00 ± 1.00^a^	0.002
Eosinophils (%)	1.00 ± 0.29	2.00 ± 0.76	1.00 ± 0.29	2.00 ± 0.76	0.442
Haematocrit (HCT, %)	33.00 ± 0.58^a^	28.40 ± 0.40^c^	32.00 ± 1.04^ab^	30.10 ± 0.62^bc^	0.007
MCV (μm^3^/cell)	96.00 ± 1.00	99.00 ± 2.08	101.00 ± 2.08	98.00 ± 1.73	0.320
MCH (pg/cell)	39.1 ± 0.87	39.00 ± 0.85	41.55 ± 0.94	42.00 ± 1.53	0.174
MCHC (%)	41.1 ± 0.70^a^	40.00 ± 0.95^b^	38.30 ± 0.44^ab^	39.00 ± 0.58^ab^	0.089
RDW-CV	14.00 ± 0.26^ab^	14.00 ± 0.42^ab^	13.10 ± 0.23^b^	15.00 ± 0.81^a^	0.133
RDW-SD	58.50 ± 0.79^a^	53.00 ± 0.81^c^	56.20 ± 0.49^b^	55.00 ± 0.51^bc^	0.003
Platelets	30.00 ± 1.00^c^	51.00 ± 1.15^b^	69.00 ± 1.53^a^	30.00 ± 1.15^c^	0.001

Values in the same row with a different superscript are significantly different (*P* ≤ 0.05). Data are presented as (mean ± SEM) of 3 replicates. * Treatments: (1) **BF**_**120**_**BC**_**0**_: The biofiltration process lasted 120 days, 60 days before and 60 days after the trial, without BC in the BF. (2) **BF**_**67**_**BC**_**67**_: Biofiltration process lasted 67 days;7 before and 60 during the experiment. The BF received 1 g/m^3^ BC every 15 days. (3) **BF**_**120**_**BC**_**60**_: Biofiltration process lasted 120 days, with 60 days without BC before the experiment. After that, the BF received 1 g BC/m^3^ media every 15 days for 60 days. (4) **BF**_**120**_**BC**_**67**_: From day 60 to day 7 before the experiment, the BF’s media was half (2.5 L) and then full (5 L). The BF was given 1 g BC/m^3^ of medium every 15 days for 67 days, starting from day 7 before the experiment. WBCs, White blood Cells, RBCS, Red blood cells, MCV, mean corpuscular volume, MCH, mean corpuscular haemoglobin, MCHC, mean corpuscular haemoglobin concentration, RDW-CV, red cell distribution width—coefficient of variation, RDW-SD, red cell distribution width—standard deviation.

### Serum biochemical analyses

#### Digestive enzymes and lipid profile

Table 8 reveals significant (*P* ≤ 0.05) differences among the tested treatments concerning the impact of the MBBR biofilter configuration on the results of the digestive enzymes and lipid profiles. The results indicated a notable increase in protease, and lipase levels, with **BF**_**120**_**BC**_**60**_ attaining the highest values relative to those of **BF**_**67**_**BC**_**67**_, which presented the lowest values. Amylase levels were comparable across treatments **BF**_**120**_**BC**_**0**_, **BF**_**67**_**BC**_**67**_, and **BF**_**120**_**BC**_**67**_ but significantly different from those in **BF**_**120**_**BC**_**60**_. The results of cholesterol, HDL-C, and HDL-C/LDL-C aligned with prior findings, favoring **BF**_**120**_**BC**_**60**_ over **BF**_**67**_**BC**_**67**_, although the serum levels of triglycerides and LDL-C were elevated at **BF**_**67**_**BC**_**67**_ relative to the other treatments.

**Table 8:** Digestive enzymes and lipid profile of Nile tilapia grown for 60 days in RAS-based tanks with diverse MBBR biofilter configurations.

Parameters	Treatments*				*P*-Value
	BF_120_BC_0_	BF_67_BC_67_	BF_120_BC_60_	BF_120_BC_67_	
Digestive enzymes					
Protease, U/L	90.00 ± 1.15^c^	77.00 ± 1.15^d^	101.50 ± 0.29^a^	94.50 ± 0.87^b^	0.001
Lipase, U/L	106.00 ± 2.89^b^	101.50 ± 2.02^b^	114.00 ± 1.15^a^	108.00 ± 1.73^ab^	0.016
Amylase, U/L	212.00 ± 0.58^b^	212.00 ± 1.73^b^	219.00 ± 1.02^a^	214.00 ± 0.58^b^	0.015
Lipid profile					
Cholesterol, mg/dL	81.50 ± 0.87^ab^	82.00 ± 1.73^ab^	85.50 ± 1.44^a^	79.00 ± 0.58^b^	0.037
Triglyceride, mg/dL	120.00 ± 1.15^c^	153.00 ± 1.15^a^	123.00 ± 0.58^c^	137.50 ± 2.02^b^	0.001
HDL-C, mg/dL	35.50 ± 0.87^b^	34.50 ± 0.87^b^	39.50 ± 0.29^a^	34.00 ± 0.58^b^	0.002
LDL-C, mg/dL	21.50 ± 0.29^b^	25.00 ± 0.58^a^	18.00 ± 0.58^c^	21.50 ± 0.87^b^	0.001
HDL-C/LDL-C	1.65 ± 0.07^b^	1.38 ± 0.06^c^	2.19 ± 0.09^a^	1.58 ± 0.09^b^	0.008

Values in the same row with a different superscript are significantly different (*P* ≤ 0.05). Data are presented as (mean ± SEM) of 3 replicates. * Treatments: (1) **BF**_**120**_**BC**_**0**_: The biofiltration process lasted 120 days, 60 days before and 60 days after the trial, without BC in the BF. (2) **BF**_**67**_**BC**_**67**_: Biofiltration process lasted 67 days;7 before and 60 during the experiment. The BF received 1 g/m^3^ BC every 15 days. (3) **BF**_**120**_**BC**_**60**_: Biofiltration process lasted 120 days, with 60 days without BC before the experiment. After that, the BF received 1 g BC/m^3^ media every 15 days for 60 days. (4) **BF**_**120**_**BC**_**67**_: From day 60 to day 7 before the experiment, the BF’s media was half (2.5 L) and then full (5 L). The BF was given 1 g BC/m^3^ of medium every 15 days for 67 days, starting from day 7 before the experiment. HDL-C, high-density lipoprotein-cholesterol; LDL-C, low density lipoprotein-cholesterol.

#### Liver and kidney function indicators

The analysis of liver and kidney function indicators demonstrated significant (*P* ≤ 0.05) variation among the tested treatments, as shown in Table 9. The results indicated that the minimum ALT value was observed in treatment **BF**_**120**_**BC**_**60**_, but the minimum AST value was noted in treatment **BF**_**120**_**BC**_**67**_. The maximum ALT and ALP values were observed in treatment **BF**_**67**_**BC**_**67**_, whereas the minimum value was observed in treatment **BF**_**120**_**BC**_**60**_. The best possible levels of urea, creatinine, uric acid, and ammonia were associated with the **BF**_**120**_**BC**_**60**_ treatment, whereas the worst values were associated with the **BF**_**67**_**BC**_**67**_ treatment.

**Table 9:** Serum liver enzymes, and kidney function indicators of Nile tilapia grown for 60 days in RAS-based tanks with diverse MBBR biofilter configurations.

Parameters	Treatments*				*P*-Value
	BF_120_BC_0_	BF_67_BC_67_	BF_120_BC_60_	BF_120_BC_67_	
Liver enzymes					
ALT, U/L	69.00 ± 0.58^b^	74.00 ± 1.15^a^	57.00 ± 1.15^c^	68.00 ± 1.73^b^	0.001
AST, U/L	136.00 ± 6.35^a^	135.50 ± 5.48^a^	127.00 ± 5.20^b^	108.50 ± 4.33^c^	0.022
ALP, mg/dL	402.00 ± 1.73^ab^	415.00 ± 2.31^a^	389.00 ± 7.51^b^	399.00 ± 0.58^b^	0.012
Kidney function indicators					
Urea, mg/dL	29.00 ± 1.15^ab^	31.50 ± 0.87^a^	24.50 ± 0.87^c^	26.50 ± 1.44^bc^	0.010
Uric acid, mg/dL	2.54 ± 0.07^bc^	3.35 ± 0.09^a^	2.36 ± 0.02^c^	2.67 ± 0.08^b^	0.001
Ammonia, mg/dL	67.00 ± 3.46^a^	67.00 ± 1.15^a^	59.00 ± 0.58^b^	67.50 ± 0.87^a^	0.037
Creatinine, mg/dL	0.79 ± 0.01^a^	0.81 ± 0.01^a^	0.42 ± 0.01^c^	0.46 ± 0.02^b^	0.001

Values in the same row with a different superscript are significantly different (*P* ≤ 0.05). Data are presented as (mean ± SEM) of 3 replicates. * Treatments: (1) **BF**_**120**_**BC**_**0**_: The biofiltration process lasted 120 days, 60 days before and 60 days after the trial, without BC in the BF. (2) **BF**_**67**_**BC**_**67**_: Biofiltration process lasted 67 days;7 before and 60 during the experiment. The BF received 1 g/m^3^ BC every 15 days. (3) **BF**_**120**_**BC**_**60**_: Biofiltration process lasted 120 days, with 60 days without BC before the experiment. After that, the BF received 1 g BC/m^3^ media every 15 days for 60 days. (4) **BF**_**120**_**BC**_**67**_: From day 60 to day 7 before the experiment, the BF’s media was half (2.5 L) and then full (5 L). The BF was given 1 g BC/m^3^ of medium every 15 days for 67 days, starting from day 7 before the experiment. GPT, glutamic pyruvic transaminase; GOT, glutamic-oxaloacetic transaminase; ALP, alkaline phosphatase.

#### Immunity, stress, and antioxidative indicators

**Fig. 3:**
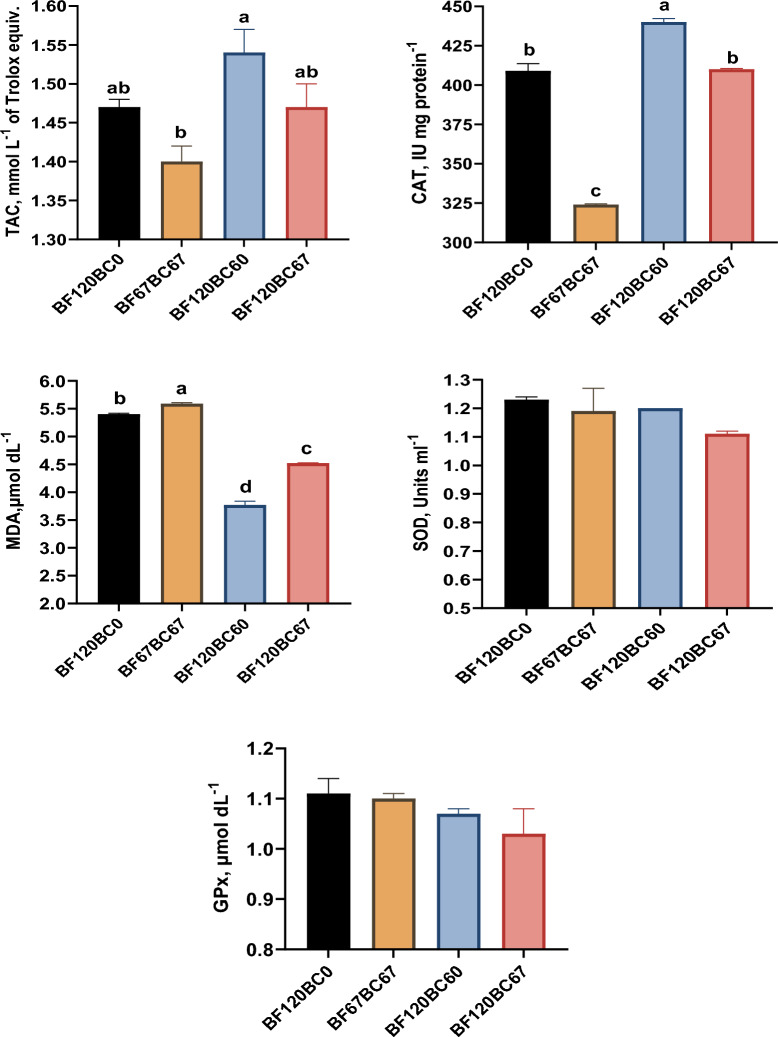
Antioxidative stress activity indicators of Nile tilapia grown for 60 days in RAS-based tanks with diverse MBBR biofilter configurations. The values with a different superscript are significantly different (*P* ≤ 0.05). Data are presented as (mean ± SEM) of 3 replicates. Treatments: (1) **BF**_**120**_**BC**_**0**_: The biofiltration process lasted 120 days, 60 days before and 60 days after the trial, without BC in the BF. (2) **BF**_**67**_**BC**_**67**_: Biofiltration process lasted 67 days;7 before and 60 during the experiment. The BF received 1 g/m^3^ BC every 15 days. (3) **BF**_**120**_**BC**_**60**_: Biofiltration process lasted 120 days, with 60 days without BC before the experiment. After that, the BF received 1 g BC/m^3^ media every 15 days for 60 days. (4) **BF**_**120**_**BC**_**67**_: From day 60 to day 7 before the experiment, the BF’s media was half (2.5 L) and then full (5 L). The BF was given 1 g BC/m^3^ of medium every 15 days for 67 days, starting from day 7 before the experiment. BC = *Bacillus spp.* consortium; SOD, superoxide dismutase; CAT, catalase; GPx, glutathione peroxidase; TAC, total antioxidative capacity; MDA, malondialdehyde.

Table 10 reveals significant (*P* ≤ 0.05) changes in serum immunity and stress indicators due to the MBBR biofilter configuration. The best serum immunity values for TP, IgM, and C3 were observed at **BF**_**120**_**BC**_**60**_, but inferior values were recorded at **BF**_**67**_**BC**_**67**_. The **BF**_**120**_**BC**_**0**_ and **BF**_**120**_**BC**_**67**_ treatments had comparable outcomes. The levels of the stress marker (cortisol) were elevated at **BF**_**120**_**BC**_**0**_ and **BF**_**67**_**BC**_**67**_, followed by **BF**_**120**_**BC**_**67**_, while the lowest value was recorded at **BF**_**120**_**BC**_**60**_. The findings of the CAT, TAC, and MDA assays demonstrated substantial variations among treatments, although the SOD and GPx tests revealed no significant changes. The best outcomes were associated with **BF**_**120**_**BC**_**60**_, whereas **BF**_**67**_**BC**_**67**_ presented the least favorable results (Fig. 3).

**Table 10:** Serum immune and stress indicators of Nile tilapia grown for 60 days in RAS-based tanks with diverse MBBR biofilter configurations.

Parameters	Treatments*				*P*-Value
	BF_120_BC_0_	BF_67_BC_67_	BF_120_BC_60_	BF_120_BC_67_	
Immune indicators					
IgM, ng/ml	10.85 ± 0.26^ab^	9.78 ± 0.48^b^	11.80 ± 0.40^a^	10.40 ± 0.52^ab^	0.052
TP, g/dL	4.04 ± 0.04^a^	3.71 ± 0.10^b^	4.08 ± 0.04^a^	4.05 ± 0.04^a^	0.005
ALB, g/dL	1.73 ± 0.02	1.73 ± 0.06	1.89 ± 0.07	1.89 ± 0.05	0.100
GLO, g/dL	2.32 ± 0.06^a^	1.98 ± 0.03^c^	2.19 ± 0.03^b^	2.17 ± 0.01^b^	0.001
ALB/GLO	0.75 ± 0.03^b^	0.87 ± 0.02^a^	0.86 ± 0.04^a^	0.87 ± 0.03^a^	0.053
C3, mg/dL	11.40 ± 0.12^b^	10.60 ± 0.23^b^	13.35 ± 0.03^a^	13.20 ± 0.64^a^	0.001
Stress indicator					
Cortisol, ng/ml	5.47 ± 0.05^a^	5.54 ± 0.06^a^	4.35 ± 0.12^c^	5.22 ± 0.02^b^	0.001

Values in the same row with a different superscript are significantly different (*P* ≤ 0.05). Data are presented as (mean ± SEM) of 3 replicates. * Treatments: (1) **BF**_**120**_**BC**_**0**_: The biofiltration process lasted 120 days, 60 days before and 60 days after the trial, without BC in the BF. (2) **BF**_**67**_**BC**_**67**_: Biofiltration process lasted 67 days;7 before and 60 during the experiment. The BF received 1 g/m^3^ BC every 15 days. (3) **BF**_**120**_**BC**_**60**_: Biofiltration process lasted 120 days, with 60 days without BC before the experiment. After that, the BF received 1 g BC/m^3^ media every 15 days for 60 days. (4) **BF**_**120**_**BC**_**67**_: From day 60 to day 7 before the experiment, the BF’s media was half (2.5 L) and then full (5 L). The BF was given 1 g BC/m^3^ of medium every 15 days for 67 days, starting from day 7 before the experiment. IgM, immunoglobulin M; TP, total protein; ALB, albumin; GLO, globulin; C3, Complement component 3.

#### Growth and immunity hormones

Table 11 illustrates the impact of various MBBR biofilter configurations on growth and immunity hormones, revealing a significant (*P* ≤ 0.05) influence on growth hormone and β-actin levels, although the treatments did not change the IGF-1 levels (*P* > 0.05). The highest concentrations of GH and β-ACTIN were detected in treatment **BF**_**120**_**BC**_**60**_, whereas the lowest values were detected in treatment **BF**_**67**_**BC**_**67**_.

**Table 11:** Growth and immunity hormones of Nile tilapia grown for 60 days in RAS-based tanks with diverse MBBR biofilter configurations.

Parameters	Treatments*				*P*-Value
	BF_120_BC_0_	BF_67_BC_67_	BF_120_BC_60_	BF_120_BC_67_	
GH, ng/ml	2.19 ± 0.02^b^	1.66 ± 0.02^c^	2.63 ± 0.03^a^	2.52 ± 0.06^a^	0.001
IGF-1, ng/ml	361.50 ± 4.33	361.00 ± 15.01	375.00 ± 7.51	370.50 ± 12.41	0.745
β-ACTIN, ng/ml	3.75 ± 0.07^ab^	3.61 ± 0.03^b^	3.87 ± 0.04^a^	3.72 ± 0.04^ab^	0.033

Values in the same row with a different superscript are significantly different (*P* ≤ 0.05). Data are presented as (mean ± SEM) of 3 replicates. * Treatments: (1) **BF**_**120**_**BC**_**0**_: The biofiltration process lasted 120 days, 60 days before and 60 days after the trial, without BC in the BF. (2) **BF**_**67**_**BC**_**67**_: Biofiltration process lasted 67 days;7 before and 60 during the experiment. The BF received 1 g/m^3^ BC every 15 days. (3) **BF**_**120**_**BC**_**60**_: Biofiltration process lasted 120 days, with 60 days without BC before the experiment. After that, the BF received 1 g BC/m^3^ media every 15 days for 60 days. (4) **BF**_**120**_**BC**_**67**_: From day 60 to day 7 before the experiment, the BF’s media was half (2.5 L) and then full (5 L). The BF was given 1 g BC/m^3^ of medium every 15 days for 67 days, starting from day 7 before the experiment. GH, growth hormone; IGF-1, insulin-like growth factor; β-ACTIN, beta-actin.

#### Histological examination of the gills, mid-intestine and liver

**Fig. 4:**
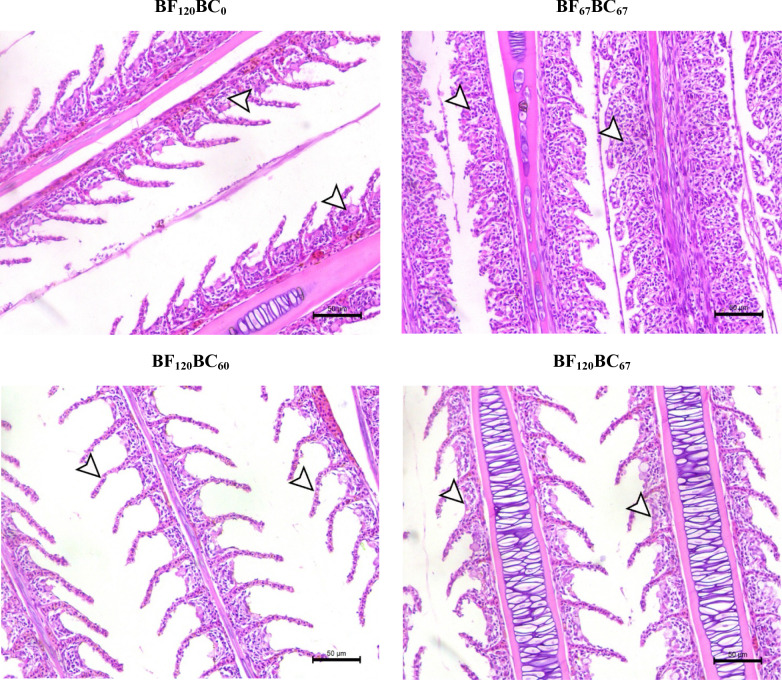
Histology of gills of Nile tilapia grown for 60 days in RAS-based tanks with diverse MBBR biofilter configurations. **BF**_**120**_**BC**_**0**_, showing moderate degree of lamellar thickening and adhesion of the secondary lamellae (arrowheads); **BF**_**67**_**BC**_**67**_, showing marked adhesion and blunting of the secondary lamellae associated with marked inflammatory cells infiltration (arrowheads); **BF**_**120**_**BC**_**60**_, showing normal long secondary lamellae (arrowheads); **BF**_**120**_**BC**_**67**_, showing mild thickening and adhesion of the secondary lamellae (arrowheads). H&E, X200 bar = 50 µm.

**Fig. 5:**
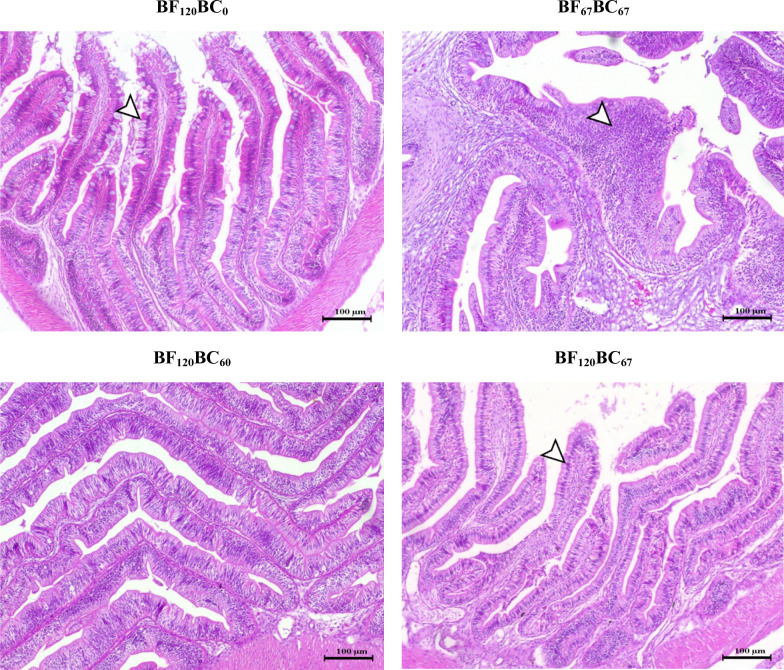
Histology of middle intestine of Nile tilapia grown for 60 days in RAS-based tanks with diverse MBBR biofilter configurations. **BF**_**120**_**BC**_**0**_, showing hyperplasia of goblets within the epithelium of the villi (arrowhead); **BF**_**67**_**BC**_**67**_, showing marked decrease of villi length with marked inflammatory cells infiltration (arrowhead); **BF**_**120**_**BC**_**60**_, showing marked increase of the length of the intestinal villi; **BF**_**120**_**BC**_**67**_, showing normal villi (arrowhead). H&E, X100, ba r = 100 µm.

**Fig. 6:**
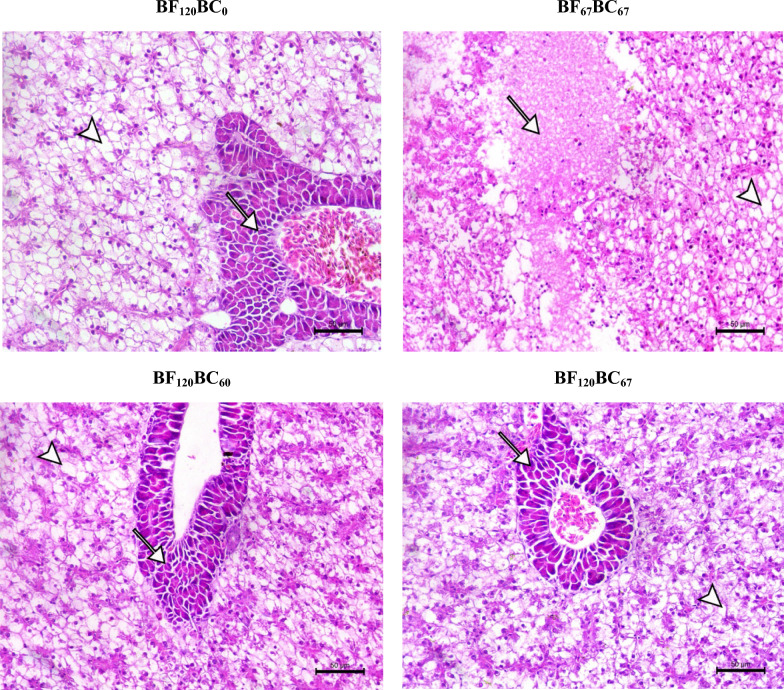
Histology of liver of Nile tilapia grown for 60 days in RAS-based tanks with diverse MBBR biofilter configurations. **BF**_**120**_**BC**_**0**_, showing mild to moderate vacuolation of the hepatocytes (arrowhead) and normal pancreatic acini surrounding central veins (arrow); **BF**_**67**_**BC**_**67**_, showing marked necrotic changes within the hepatopancreatic tissue associated with severe oedema (arrow) and marked vacuolation of the hepatocytes (arrowhead); **BF**_**120**_**BC**_**60**_, showing normal hepatopancreatic tissues with mild vacuolation of the hepatocytes (arrowhead) and normal pancreatic acini surrounding central veins (arrow); **BF**_**120**_**BC**_**67**_, showing mild vacuolation of the hepatocytes (arrowhead) and normal pancreatic acini surrounding central veins (arrow). H&E, X200 bar = 50 µm.

Figure 4 shows the results of the histological examination of the gills. The results of the histological investigation of the intestine (middle portion) are displayed in Table 12 and Fig. 5, whereas the histopathological examination of the liver is illustrated in Fig. 6. The midgut histological findings indicated that the villus length in **BF**_**120**_**BC**_**60**_ was approximately threefold greater than that in **BF**_**67**_**BC**_**67**_, whereas the number of goblet cells in **BF**_**120**_**BC**_**60**_ was roughly fourfold greater than that in **BF**_**67**_**BC**_**67**_. The outcomes for **BF**_**120**_**BC**_**0**_ and **BF**_**120**_**BC**_**67**_ treatments were highly comparable. According to Fig. 4, **BF**_**120**_**BC**_**0**_ has some thickening and sticking of the secondary lamellae; **BF**_**67**_**BC**_**67**_ has many sticking and flattening of the secondary lamellae along with many inflammatory cells; **BF**_**120**_**BC**_**60**_ has normal long secondary lamellae; and **BF**_**120**_**BC**_**67**_ has slight thickening and sticking of the secondary lamellae. Figure 5 shows that **BF**_**120**_**BC**_**0**_ has more goblet cells in the villi epithelium, **BF**_**67**_**BC**_**67**_ has shorter villi with many inflammatory cells, **BF**_**120**_**BC**_**60**_ has longer intestinal villi, and **BF**_**120**_**BC**_**67**_ has normal villi. Figure 6 shows that **BF**_**120**_**BC**_**0**_ shows mild to moderate changes in liver cells and normal pancreatic architecture adjacent to central veins; **BF**_**67**_**BC**_**67**_ reveals severe damage to liver and pancreatic tissues, marked by substantial edema and significant alterations in liver cells; **BF**_**120**_**BC**_**60**_ displays normal liver and pancreatic tissues with minor alterations in liver cells and preserved pancreatic structures around central veins; and **BF**_**120**_**BC**_**67**_ indicates slight changes in liver cells while maintaining normal pancreatic structures around central veins.

**Table 12:** Middle intestine histological examination of Nile tilapia grown for 60 days in RAS-based tanks with diverse MBBR biofilter configurations.

Parameters	Treatments*				*P*-Value
	BF_120_BC_0_	BF_67_BC_67_	BF_120_BC_60_	BF_120_BC_67_	
Villi length (µm)	421.41 ± 3.22^b^	197.79 ± 14.53^c^	646.46 ± 8.37^a^	435.88 ± 8.00^b^	0.001
Villi width (µm)	92.08 ± 7.09^b^	167.37 ± 10.35^a^	81.07 ± 2.88^b^	86.67 ± 2.81^b^	0.001
Intervilli distance (µm)	79.87 ± 4.91^b^	107.06 ± 4.56^a^	45.73 ± 2.37^d^	67.67 ± 1.93^c^	0.001
Goblet cells (#/mm^2^)	111.33 ± 5.21^c^	57.00 ± 4.16^d^	225.00 ± 6.03^a^	130.00 ± 4.16^b^	0.001

Values in the same row with a different superscript are significantly different (*P* ≤ 0.05). Data are presented as (mean ± SEM) of 3 replicates. * Treatments: (1) **BF**_**120**_**BC**_**0**_: The biofiltration process lasted 120 days, 60 days before and 60 days after the trial, without BC in the BF. (2) **BF**_**67**_**BC**_**67**_: Biofiltration process lasted 67 days;7 before and 60 during the experiment. The BF received 1 g/m^3^ BC every 15 days. (3) **BF**_**120**_**BC**_**60**_: Biofiltration process lasted 120 days, with 60 days without BC before the experiment. After that, the BF received 1 g BC/m^3^ media every 15 days for 60 days. (4) **BF**_**120**_**BC**_**67**_: From day 60 to day 7 before the experiment, the BF’s media was half (2.5 L) and then full (5 L). The BF was given 1 g BC/m^3^ of medium every 15 days for 67 days, starting from day 7 before the experiment.

## Discussion

Bacteria are essential in metabolizing ammonia and other metabolites during the biofiltration process within recirculating aquaculture systems (RASs)^56^. Among the many probiotic species discovered, *Bacillus* species have been proven to combat fish pathogens effectively because of their ability to produce antimicrobial substances^57^, improve immune responses, feed utilization, digestive enzyme activities, digestibility, and water quality^58,59^. Heterotrophic and autotrophic bacteria maintain balance at the system level as long as the C/N ratio does not drastically change^60–62^ and autotrophs do not suffer from oxygen limitation^63^. In this research, a small-scale RAS was designed that can be used as a solution to develop a sustainable aquaculture production system. The biofiltration process was performed via a moving bed biofilm reactor (MBBR) with special commercial media (PE03). The use of MBBR in biofiltration is recommended for high-efficiency small-scale RASs^5,64,65^. Sanolife (a commercial probiotic) containing a *Bacillus spp*. consortium (BC) was added in this study to biofilters with diverse configurations (different timings of addition). The Sanolife probiotic was applied to feed and/or added to water^66^. They are nonpathogenic and nontoxic microorganisms without undesirable side effects when administered to aquatic organisms^67^.

### Water quality

The most harmful water quality issues encountered in RASs have been linked to both low dissolved oxygen and elevated levels of metabolic waste, including total ammonia nitrogen, free ammonia, nitrite, and carbon dioxide^68^. The results of the present study revealed no significant differences in water quality metrics but did reveal significant changes in pH and alkalinity values. Severe fluctuations in the TAN, NH₃, NO₃, and NO₂ values in **BF**_**67**_**BC**_**67**_ were evident during the first ten days, in contrast with the relative stability of the other coefficients. The lowest TAN value was recorded for **BF**_**120**_**BC**_**60**_. The results of this study are consistent with the results of other researchers^5,69^. The rationale for the enhanced water analysis results, particularly in treatment **BF**_**120**_**BC**_**60**_, is attributed to the two-month preparation period of the biological filter prior to the experiment, alongside the continuous inoculation of *Bacillus spp.* consortium throughout the duration, in contrast to the short seven-day preparation period applied in treatment **BF**_**67**_**BC**_**67**_. The preparation time of a biological filter is crucial for its optimal performance. Sufficient time allows beneficial bacteria to colonize the filter media, forming a stable biofilm. The acclimatization period required for stable biofilm formation varies significantly on the basis of the conditions, ranging from 7 to 42 days in freshwater biofilters^69,70^, whereas the natural colonization of nitrifying biofilms in saltwater biofilters may take several days, specifically 28–81 days^71^. The natural activation of bacteria in biofilters or the addition of concentrated bacteria may serve as expedient solutions. An effective biofilter activation technique requires the swift proliferation of nitrifying bacteria capable of oxidizing ammonium to nitrite and, subsequently, to nitrate while maintaining nitrite concentrations below dangerous thresholds^27,72^. These beneficial microorganisms are essential for the transformation of harmful nitrite into nontoxic nitrate^27^.

The colonization of biofilm carriers by nitrifying bacteria is often time-consuming due to the generally slow growth rates of these bacteria^73,74^. Ammonia-oxidizing bacteria (AOB) and nitrite-oxidizing bacteria (NOB) exhibit slow growth, and their activity is directly enhanced by substrate availability^75^, indicating that both ammonium and nitrite are essential for rapid biofilm initiation^76^. Efficient activation methods for rapid biofilters are crucial for sustainability, economic viability, and welfare considerations in RASs^77^. The ideal activation method involves the rapid enrichment of nitrifying bacteria in the biofilter before the stocking of postsmolt salmon; nevertheless, knowledge regarding the exact microorganisms to include in an RAS biofilter inoculum is still inadequate^77^. Zhang, et al.^78^ revealed that incorporating *B. licheniformis* bacteria into rearing water reduces the concentrations of harmful substances (TAN and NH_3_). The inclusion of *Nitrosomonas* and *Nitrobacter* probiotics markedly reduces nitrite levels and elevates nitrate levels in Nile tilapia cultivated in an aquaponic system, with 1.5 mg/L identified as the optimal dosage^79^. *Bacillus* species accelerate the biodegradation of nitrogenous wastes, leading to waste mineralization^78^. The significant proliferation of bacteria might adversely impact fish by functioning as pathogens or competing for oxygen^80,81^. However, a prior study demonstrated that the introduction of a low-density fish population into a novel recirculating aquaculture system is the most efficacious approach for biofilter activation^82^. Elevated alkalinity in **BF**_**120**_**BC**_**60**_ is frequently correlated with increased biofiltration activity, as indicated by the higher pH in **BF**_**120**_**BC**_**60**_ than in **BF**_**67**_**BC**_**67**_. The elevated alkalinity in the RAS water may result from the denitrification process. Denitrification is the process by which bacteria transform nitrates into nitrogen gas, resulting in the release of hydroxide ions that increase alkalinity^83^. The level of alkalinity recorded in this study is consistent with that reported in many previous studies. Maintaining sufficient alkalinity levels is essential for nitrification. Chen, et al.^84^ recommended maintaining an alkalinity level of 200 mg CaCO3 L^−1^ in aquaculture to facilitate nitrification. Summerfelt^28^ proposed an alkalinity of 70 mg CaCO3 L^−1^, but a broader range of 50–300 mg CaCO3 L^−1^ has been reported^81^.

Pathogenic diseases in RAS aquaculture are often linked to bacteria such as *Aeromonas*, *Vibrio*, *Streptococcus*, *Yersinia*, *Acinetobacter*, *Lactococcus*, *Pseudomonas*, and Clostridium^85^. Bacillus species, such as *Bacillus subtilis* and *Lactobacillus acidophilus*, can enhance the immune response, resistance, and growth performance of Nile tilapia by competing with natural pathogenic microorganisms^86^. The present study demonstrated the absence of pathogenic bacteria (*Vibrio spp.* and *Aeromonas spp.*) in the aqueous media of the different treatments. The results suggest that the rearing water has a healthy microbial balance, which is the main goal of efficient RAS management and could be achieved through the use of an established biofilter and the addition of probiotics. Another possible reason for the absence of pathogenic bacteria may be the overall hygiene of the system, the quality of the municipal water (which is free of harmful pathogens), the quality of the fish, and the efficient use of high-quality, pathogen-free feed, all of which contribute to maintaining a safe and healthy environment for aquatic life. In the aquatic environment, poor water quality encourages the growth of pathogenic microbes that can spread to aquatic species^87^. The use of municipal water in lab/small-scale RAS fish farming has many benefits, especially in terms of harmful microbial load. This beneficial effect is due to municipal water safety being ensured by chlorine, which effectively inactivates a wide range of microorganisms, including harmful bacteria and viruses, rather than surface water or underground water^88^. Although there are significant differences in the concentration of *Streptococcus spp.* and total bacterial count among the tested treatments, they are still much lower than the limits that adversely affect the efficiency of the RAS system. In tilapia aquaculture systems, *Streptococcus spp.* levels in the water column that exceed 10^3^ to 10^4^ CFU/mL (colony-forming units per milliliter) are considered a significant risk factor for initiating *streptococcosis* outbreaks^89^.

### Growth performance, feed utilization, and chemical composition

The intricate bacterial community in RAS biofilters is vital, with nitrifying bacteria being important for maintaining the optimal water quality required for healthy fish growth^77^. The results of the present study revealed significant changes in growth performance, feed efficiency, and biometric measurements among the treatments. The **BF**_**120**_**BC**_**60**_ treatment had the best growth and feed utilization metrics, whereas the **BF**_**67**_**BC**_**67**_ treatment had the poorest growth and feed utilization metrics. Nguyen, et al.^90^ reported that, in the RAS, biofilter efficiency, the fish growth rate, and nutritional utilization are closely interconnected. A well-functioning biofilter is important because it preserves water quality by removing toxic ammonia, which directly impacts fish health and growth. Efficient biofiltration leads to better feed utilization, as fish are less stressed and can convert feed into growth more effectively. Inoculating a biofilter in an RAS with beneficial bacteria can significantly improve filtration efficiency, leading to better water quality and ultimately enhancing fish growth and nutrient utilization^91^. Adding specific beneficial bacteria to pond water, particularly in systems with low/zero water change rates, can positively improve growth rates, increase nutritional utilization, and even increase protein content in fish^59,92,93^. The results of the present study revealed no significant differences in dry matter, fat, ash, or energy content. **BF**_**67**_**BC**_**67**_ had the lowest protein level, while the other three treatments had similar protein levels. Moreover, the proteolytic activity of Bacillus enhances protein availability and the absorption of amino acids and vitamins^94^. The results of HSI and VSI in aquaculture studies are important indices of fish physiology and energy stores. The hepatosomatic index (HSI), which compares liver size to body weight, reflects the liver’s metabolic status and adaptive responses to water quality/dietary changes^95^. The reduced HSI in BF_67_BC_67_ indicates hepatic damage in histology as illustrated in Fig. 6 and may be correlated with the high values of TAN and NH₃ in the rearing water. The hepatic health status in **BF**_**67**_**BC**_**67**_ negatively increased the values of liver enzymes (ALT, ALP) as indicated in Table 9. In a healthy, well-fed, non-polluted environment, a moderate increase in HSI might simply indicate high energy storage^96^. Besides, the** e**xposure to toxins or poor water conditions (like high ammonia) can, in some cases, result in abnormally low HSI. A reduced HSI, often accompanied by histopathological damage, indicates poor liver function and reduced energy reserves in tilapia^97^. The elevated VSI values in treatment **BF**_**67**_**BC**_**67**_ may be attributed to an increase in the fat content of the digestive tract, as evidenced by the highest fat content (19.50%) observed in the chemical analysis of the fish’s body, as well as elevated blood lipid levels (triglycerides and LDL-C). In high-intensity systems, poor water quality, specifically low dissolved oxygen and high ammonia, acts as a chronic stressor. When fish are stressed, their metabolism shifts. Instead of using nutrients for muscle protein synthesis, they often redirect energy toward lipid storage in the visceral cavity^98^.

### Blood hematological analysis

Blood examination reveals information about various aspects of fish health, including their oxygen-carrying ability (hemoglobin, hematocrit), immune response (white blood cell count), and overall physiological status^99^. Hematobiochemical variables serve as straightforward and effective diagnostic tools to elucidate the immune response and resistance to infections in many aquatic species^100^. The current study revealed significant differences in the blood analysis results among the treatment groups, with **BF**_**120**_**BC**_**0**_, **BF**_**120**_**BC**_**60**_, and **BF**_**120**_**BC**_**67**_ resulting in higher hemoglobin levels and **BF**_**67**_**BC**_**67**_ resulting in lower values. The WBC numbers were lower at **BF**_**120**_**BC**_**60**_, and the hematocrit levels were significantly different, with **BF**_**67**_**BC**_**67**_ having the lowest values. Unfortunately, to the best of our knowledge, no research has investigated the influence of biofiltration configuration on the quality of blood metrics in fish. Biofiltration configurations in aquaculture systems, including the addition of probiotics in low/zero water exchange, significantly impact fish blood quality by improving water quality, which in turn affects various hematological parameters. Specifically, biofilters reduce harmful ammonia and nitrite levels, leading to healthier fish with improved blood profiles. Such improvements include positive changes in red blood cell (RBC) counts, hemoglobin (Hb) levels, and hematocrit (HCT) levels^93^. The biofiltration process helps remove harmful pollutants and waste products, creating a cleaner environment that supports better physiological function in fish^101^. Elevated levels of white blood cells (WBCs) and other stress markers in fish blood can indicate exposure to toxins and disease. By improving water quality, effective biofiltration can help reduce these stress responses^102^. In general, introducing beneficial bacteria into the aquatic environment is expected to improve indicators of fish blood quality. The quality of the aquatic environment directly influences the blood parameters, serum analytical parameters, and well-being of cultured organisms^59,93,103^.

### Serum liver enzymes and kidney function indicators

The design and operation of recirculating aquaculture systems (RASs) affect fish health, since parameters such as stocking density, water quality, and feed management influence stress and immunological responses. Liver enzymes and kidney function indicators serve as biomarkers for evaluating fish health^104^. The reduced levels of liver enzymes and kidney function indicators in Nile tilapia at **BF**_**120**_**BC**_**60**_ in this study demonstrated a favorable correlation between a healthy environment and the well-being of the liver and kidneys. Increased concentrations of liver enzymes such as ALT and AST, in conjunction with markers of kidney function such as urea and creatinine, may indicate stress, disease, or poor water quality resulting from elevated CO₂ levels or the presence of pollutants^105,106^. El-Kady, et al.^93^ reported that the addition of probiotics in closed tilapia tanks significantly reduced the levels of ALT, AST, and urea. The reduced cortisol levels in the **BF**_**120**_**BC**_**60**_ treatment relative to those in the other treatments often indicate a more favorable rearing environment. Increased cortisol levels may correlate with physiological stress responses due to inadequate water quality^107^. Abdel-Rahim, et al.^59^ reported that the addition of probiotics to low water exchange seabass (*Dicentrarchus labrax*) tanks significantly reduced cortisol levels.

### Serum immunity and antioxidant parameters

RAS can affect immune parameters such as lysozyme, immunoglobulin, and antioxidant enzyme activity in fish, which can be measured in serum samples. Changes in serum immunity parameters can indicate a fish’s response to environmental stressors and potential disease outbreaks in RASs^104^. The increased immunity and antioxidant levels in the **BF**_**120**_**BC**_**60**_ treatment increased in the tilapia growing environment relative to those in the other treatments, particularly **BF**_**67**_**BC**_**67**_. The elevated blood protein profile (total protein, albumin, and globulin) signifies an improvement in humoral immunity linked to augmented systemic immunity during illness^25^. Adding specific bacteria to biofilters can increase their efficiency and positively impact the immune system of the fish, as reflected in serum parameters^108^. The use of probiotics in closed or semi-closed aquaculture systems, whether freshwater or saltwater, significantly enhances immunity and antioxidant resilience due to the enhanced environmental conditions and health of the fish^59,93^. Probiotics, especially *Bacillus sp*., exhibit metabolic activity that eradicates oxidative compounds, modulates tissue damage, and enhances the synthesis of antioxidant enzymes such as SOD and glutathione^109,110^. They contribute to antioxidant defense, balance, and oxidative stress in fish by diminishing the generation of reactive oxygen species (ROS)^111^. These antioxidant defense systems are stimulated and connected to the host’s response to pathogenic infections, resulting in stable physiological responses in fish without hydrogen peroxide release during β-oxidation^112^.

### Serum digestive enzymes and lipid profile of Nile tilapia

The results of the present study demonstrated that optimizing the tilapia welfare environment in the **BF**_**120**_**BC**_**60**_ treatment positively influenced the concentration of digestive enzymes and the lipid profile relative to those in the other treatments. Regrettably, there are no prior studies addressing the correlation between biological filter configurations and both digestive enzyme and lipid profiles. The efficiency of fish digestive enzyme activity directly correlates with the optimization of their aquatic environment^103,113^. Beneficial bacteria facilitate the synthesis of digestive enzymes such as amylases, proteases, and lipases, which increase the absorption of amino acids, fatty acids, and vitamins (including B complexes and folic acid), thereby promoting increased growth^61,94^. *Bacillus subtilis* and *Bacillus cereus* are utilized as probiotics for fish, enhancing growth and nutritional assimilation by increasing digestive enzyme activity and strengthening the immune system^114^. The inclusion of probiotics in culture water enhances digestive enzymatic activity, resulting in improved feed digestibility^93^. Tomaro-Duchesneau, et al.^115^ reported that probiotic bacteria facilitate energy absorption and can decrease fat levels, thereby increasing lipid processing in fish by increasing nutrient absorption, retention, and utilization by cells, resulting in decreased blood fat levels^112^. Improving the environment improves feed fat utilization and the blood lipid profile. El-Dahhar, et al.^62^ reported that improving the water quality of biofloc red tilapia significantly reduced cholesterol, triglyceride, and LDL levels while increasing HDL.

### Growth and immunity hormones

The advantages of mitigating oxidative stress are evident in animal growth and homeostasis, as the liver is the principal organ responsible for synthesizing growth hormones, whereas hepatocytes facilitate the regulation of nutrient metabolism and the excretion of harmful substances^116^. The GH/IGF-I axis plays a crucial role in regulating physiological responses in fish, including growth regulation^117^. GH secretes IGF-I, which acts as a mediator of GH actions in regulating somatic growth^118^. Compared with those in the other treatments, the levels of the hormones GH, IGF-1, and β-ACTIN in the current study were greater in the **BF**_**120**_**BC**_**60**_ treatment. This finding is consistent with several studies linking the levels of growth/immune-related hormones to stress factors such as fish density in RAS systems^104^, pollutants^119^, and improving environmental quality by supplementing the water with probiotics^93^. As in the present study, other studies have indicated that cortisol can reduce plasma levels and transcription of GH and IGF, leading to growth suppression in fish under stress^120^. In tilapia, cortisol can inhibit both plasma levels and transcription in the liver of IGF-I. These findings suggest that cortisol can interact with the GH/IGF-I axis, potentially inhibiting both GH and IGF-I secretion in teleost species under stressful situations^104,121^. Numerous studies have indicated that the administration of probiotics significantly enhances IGF-I expression in fish^122,123^. Growth and immunity hormones are chiefly affected by genetics, nutrition, and environmental conditions, with an indirect effect on the bacterial composition of biofilters^124^. The increased quality and quantity of bacteria introduced to water or biofilters correlate with elevated quantities of hormones linked to growth and immunity^93^. Gene expression analysis is essential for elucidating the impact of RAS management measures on fish health, disease resistance, and system performance, with β-actin serving as a reference gene, which is vital to this process^125^.

### Gills, intestine and liver histopathological examination

The health of internal organs, such as the gills, intestines, and liver can provide a more precise estimate of fish health than can morphometric measurements and blood analysis. It also affects the environmental health of both closed and open waters^93^. Gill health can be assessed by observing the fish for signs of damage, such as excessive mucus production, pale or eroded gill filaments, and difficulty breathing^126^. The slow development of nitrifying bacteria can lead to elevated amounts of ammonium and nitrite in immature biofilters. Free ammonia can harm gills and internal organs and osmoregulation in fish, and the balance between ammonia and ammonium in water is influenced primarily by pH^27^. In recirculating aquaculture systems (RASs), high levels of TAN, NH₃, and NO₂ can severely impact fish gill health, leading to damage, reduced oxygen uptake, and even mortality^127^. The present study revealed significant differences in gill health, with **BF**_**120**_**BC**_**60**_ being better than the other treatments. The explanation is primarily attributable to the superior cleanliness of this treatment. Poor gill health can lead to reduced growth, increased susceptibility to diseases, and, in severe cases, mortality^128^.

In the intestine, significant health improvements, especially in terms of villus length and goblet cell numbers, were recorded in favor of **BF**_**120**_**BC**_**60**_ compared with the other treatments. The intestine histology results support the previously mentioned findings concerning digestive enzymes levels, nutrient utilization and growth reported in this study. Also, this finding is supported by several studies showing that *Bacillus spp.* can settle in the fish gut, which helps produce organic acids, activate digestive enzymes, and remove harmful substances from their food, leading to a healthier gut and better nutrient digestion and absorption^129,130^. Recently, *Bacillus* was shown to displace pathogenic bacteria from the gut, increasing disease resistance and improving fish performance^66,131^. The height of the intestinal villi, thickness of the muscular layer, and number of goblet cells are exemplary indicators of intestinal health^132^. The larger surface area of the intestines, which helps absorb more nutrients, along with the greater number of goblet cells, explains the better growth, stronger immune system, and improved ability to handle stress in Nile tilapia^66^. The increase in the area of the intestinal villi absorbed by the probiotic bacteria in this study^112^ signifies the beneficial effects of the additive, which positively influence the integrity of the intestinal mucosa and nutrient assimilation^133^ while also promoting homeostasis in the animals^112^. In RASs, the health of the fish liver is a valuable indicator of overall water quality and the health of the aquatic environment. Monitoring liver health can help optimize RAS management and ensure the well-being of farmed fish^134,135^. Compared with fish in other systems, those raised in the RAS can exhibit changes in liver metabolism and antioxidant capacity. Therefore, including liver health assessments in the monitoring program of an RAS is a valuable tool for ensuring the sustainability and success of aquaculture operations^136^.

## Conclusion

Biological filters are essential for the efficacy and success of recirculating aquaculture systems (RASs). This study aimed to assess four moving bed biofilm reactors (MBBR, abbreviated as BF). configurations in terms of preparation time before the operational fish production cycle and to examine the influence of the introduction of *Bacillus spp*. consortium on water purification and its effects on fish performance, health, production, and sustainability. The results indicated that the performance of a biological filter is directly proportional to its preparation duration; prolonged preparation time increases the efficiency of the biofiltration process. Furthermore, introducing beneficial bacteria during and subsequent to the biological filter preparation period, as well as frequently thereafter, enhances its efficacy and filtration performance. The **BF**_**120**_**BC**_**60**_ treatment, which included a preliminary two-month biofilter preparation followed by an additional two months of probiotic (*Bacillus spp.* consortium) supplementation during the 60-day experiment, produced the most favorable results among all treatments evaluated. The **BF**_**120**_**BC**_**60**_ treatment led to notable enhancements in various aspects, including water quality, growth rate, and nutritional efficiency. Additionally, it improved blood and serum health indicators, which include digestive enzymes, immune parameters, antioxidant indices, and growth/immune-related hormones. Furthermore, the treatment positively affected the condition of internal organs, including the gills, intestine, and liver.

## Data Availability

No datasets were generated or analysed during the current study.
